# Area-Based Socioeconomic Position and Adult Glioma: A Hierarchical Analysis of Surveillance Epidemiology and End Results Data

**DOI:** 10.1371/journal.pone.0060910

**Published:** 2013-04-09

**Authors:** Jesse J. Plascak, James L. Fisher

**Affiliations:** 1 Ohio State University Comprehensive Cancer Center, James Cancer Hospital and Solove Research Institute, Columbus, Ohio, United States of America; 2 Division of Epidemiology, College of Public Health, The Ohio State University, Columbus, Ohio, United States of America; University of Navarra, Spain

## Abstract

**Background:**

Glioma rates vary by demographic factors and geo-political boundaries and this variation suggests higher glioma rates in groups of higher socioeconomic position. The primary goal of this analysis is to investigate the relationship between glioma and county socioeconomic position using U.S. Surveillance Epidemiology and End Results (SEER) data.

**Methods:**

Cases were individuals 25+ years diagnosed with glioma between 2000 and 2006 and residing within the SEER-17 catchment area. County-, sex-, race-, age-specific rates were created in order to investigate individual-level associations (population data from U.S. Census 2000). A Bayesian hierarchical Poisson spatial conditionally autoregressive (CAR) model was utilized to simultaneously estimate individual- and county-level associations while controlling for county spatial dependence.

**Results:**

Those residing in counties of the second, third, and fourth highest quartiles of socioeconomic position have glioma incidence rates that are 1.10 (95% CI: 1.02,1.19), 1.11 (95% CI: 1.02,1.20), 1.14 (95% CI: 1.05,1.23) times that of the first quartile, respectively. A CAR model properly controlled for error spatial dependence. Investigated lag times suggest year 2000 census data yields superior model fit.

**Conclusion:**

Demographically adjusted rates of glioma are elevated in counties of higher socioeconomic position. More well-grounded theory concerning the glioma-socioeconomic position association along with socioeconomic data collected at multiple levels is recommended for future studies investigating this relationship.

## Introduction

Glioma comprise approximately 80% of all primary malignant brain and central nervous system tumors in the U.S. [1]. The five-year survival probability of individuals diagnosed with glioma varies by subtype, but is as low as 4.7% [1]. Little is known about the etiology of glioma, with ionizing radiation and family history being the only identified non-genetic risk factors [2].

Data from cancer registries in the U.S. suggest that rate differences exist across socio-demographic groups and geopolitical boundaries [1]. The 2005–2009, average annual, age-adjusted incidence rate of glioma was 7.2 (per 100,000 person-years) among males and 5.1 among females, and the rate varied considerably by race (at least two fold increase comparing whites to blacks across several glioma subtypes) [1]. A study of glioblastoma multiforme, the dominant and largely fatal glioma subtype, revealed similar sex and race differences while also demonstrating higher rates among those residing in high socioeconomic areas (rate ratio (RR) = 1.3, 95% confidence interval (CI) 1.2,1.4), even after statistical adjustment for confounding factors [3]. A recent Swedish study reported an increased odds of glioma among those with a higher family income (odds ratio (OR) 1.5, 95% CI: 1.1, 2.1), adjusted for sex, age, and geographic region [4].

Previous studies note possible associations between brain tumor risk and occupations related to certain levels of individual socioeconomic position (SEP); however, the direction and magnitude of these associations vary greatly. Grayson reported an increased odds of glioma among U.S. Air Force officers compared to enlistees (OR = 2.1 95% CI: 1.5, 3.0) [5]. In another study, although adult glioma risk was not associated with most white-collar occupations, physicians, and legal and social service workers had increased odds of glioma; OR and 95% CIs for physicians and legal/social service workers, respectively, 3.4 (1.1, 11.1) and 2.4 (1.0, 6.0) [6]. However, blue-collar occupations were more frequently associated with increased glioma odds.

This analysis is designed to study the relationship between glioma and indicators of socioeconomic well-being in the U.S. A multilevel framework is utilized to enable the proper estimation of group-level – together with individual-level – associations with glioma in a single model [7]. The primary aim is to assess the glioma burden across race, sex, and age groups while determining whether or not glioma risk is associated with area-based SEP.

## Materials and Methods

### Data Description

Data were gathered from the Surveillance Epidemiology and End Results (SEER) Program of the National Cancer Institute, which, for the time period of these analyses, collected information on all invasive tumors diagnosed among residents of 17 U.S. regions [8]. These regions covered 28% of the U.S. population.

Glioma diagnoses were classified by histology codes into groupings using the International Classification of Disease – Oncology – Version 3 (ICD-O-3) [9]. ICD-O-3 histology diagnostic codes ‘9380’ thru ‘9489’ were identified as glioma (see Table 1 for detailed histologic groupings and codes). Cases were defined as individuals 25+ years diagnosed with glioma between 2000 and 2006 and residing within the SEER 17 catchment area. County-, sex-, race-, age-specific rates were created to investigate individual-level associations nested within counties (population data from U.S. Census 2000). Alaskan registry cases were excluded because of the vast difference in spatial scale between Alaska and other SEER counties. Louisiana registry cases were not reported from July through December 2005 due to hurricane Katrina; therefore, all Louisiana cases were excluded because of this temporal mismatch. Subgroupings registering zero population during the study period were also excluded (N = 237). The final subgroup sample size was 7,035 (404 counties, 2 sexes, 3 races [white, black, other], and 3 age groups [“young adult”/25–44 yrs, “middle-age”/45–64 yrs, “Elderly”/65+ yrs]). (Too few cases among non-white, non-black cases prohibited analyses of specific additional race groups.).

**Table 1:** Histologic breakdown of glioma cases diagnosed within the SEER Program (17 registries), 2000–2006.

Histology (ICD-O-3 Histology Code^a^)	Column %
Glioblastoma (9440, 9441, 9442/3)	51.0%
Astrocytoma, NOS (9400)	7.9%
Glioma, NOS (9380)	7.1%
Anaplastic astrocytoma (9401, 9411)	7.0%
Pilocytic astrocytoma (9421)	5.4%
Oligodendroglioma (9450)	5.4%
Ependymoma/anaplastic ependymoma (9391–9394)	4.2%
Embryonal/primitive/medulloblastoma (8963, 9363, 9364, 9470, 9471, 9472, 9473, 9474, 99501, 9502, 9503, 9508)	3.6%
Mixed glioma (9382)	3.1%
Anaplastic oligodendroglioma (9451, 9460)	2.4%
Diffuse astrocytoma (protoplasma, fibrillary) (9410, 9420)	1.4%
Not Brain	0.7%
Unique astrocytoma variants (9383, 9384, 9424)	0.4%
Neuroepithelial (9381, 9423, 9430, 9444)	0.3%
Choroid plexus (9390)	0.2%

a International Classification of Disease – Oncology – Version 3 [9].

County-level SEP data were obtained from SEER (as included in SEER*Stat software and as originally collected from U.S. censuses of population, 1990 and 2000) and linked to the county- and demographic-specific rates. SEP data included: median household income, percent of population with less than a high school education by age 25 years, percent of population below 100% of the federal poverty level (FPL), percent of population unemployed, percent of population foreign born, and percent of population residing in urban portions of a county. These six county-level demographic variables were gathered for both 1990 and 2000 U.S. census years.

### Statistical Analysis

Principal Component Analysis (PCA) of the SEP-related variables was utilized to reduce data dimensionality and eliminate possibility of collinearity between county covariates [10]. PCA analysis proceeded using a data correlation matrix and only components with Eigenvalues greater than 1.0 were considered in subsequent modeling. ‘Varimax’ (orthogonal) rotation was chosen ensuring zero correlation between factors [11]. *Post hoc* natural log transformation was performed for all variables except percent urban to improve normality of the resulting score distribution. These standardized components were used in subsequent modeling.

A Bayesian multilevel framework with random intercept parameters was used to allow for differences in subgroup glioma rates between counties. This also enabled the ability to model the county-level random effects as a function of the SEP components in a second level. Subgroup rates were assumed to conditionally follow a Poisson distribution. The response was related to the explanatory variables through a log link function (see Equation S1 for model notation).

Two basic models were sequentially built – one including individual-level subgroupings and county random intercepts with SEP components as covariates in a second level (hereafter, referred to as ‘Model 1’), and another following model 1 while spatially structuring the county random intercepts following a conditionally autoregressive (CAR) prior distribution (hereafter, referred to as ‘Model 2’) [12], [13]. Estimates of the county variables involved in the creation of the SEP composite were also independently examined to allow comparisons to previous literature. The assumption of conditional independence was examined for each model by analyzing county rate error using Moran’s I. Type-I error of 0.05 was *a priori* chosen for Moran’s I, while estimate’s 95% CIs and model Deviance Information Criterion (DIC) were used in decision making for the Bayesian model-building. Those covariates with 95% CIs including the null (*H_0_*_:_ *β_n_ = *0*)*, or not resulting in DIC decreases (compared to a model containing everything except the examined covariate) were excluded from the final model.

Time lag between SEP and glioma was investigated by constructing separate models using PCA derived components from 1990 and 2000 census variables [14], [15]. Appropriate lag was informed by comparing model fit via the DIC of the two models. Cross-level SEP effect modification was assessed by stratifying the final dataset (N = 7,035) on SEP quartiles and running separate models within each stratum. Changes in individual-level median coefficients were inspected visually and 95% CIs were compared across strata. An individual-level covariate was deemed a confounder if the following two conditions were met: 1) there was a change of at least 10% in the SEP coefficients resulting from removal of the potential confounder from the model, and 2) the potential confounder was associated with both glioma and SEP.

Flat prior distributions were assigned to fixed-effect regression coefficients [i.e., coefficients ∼ *Normal* (0, 1^−10^)] and the variance of the random effect parameter [random effect parameter variance *∼* gamma (0.001, 0.001)]. Relative to the precision of the data, specifying small precisions for these prior distributions allowed the resulting estimates to be minimally influenced by these priors (see Gelman for details) [16]. Markov Chain Monte Carlo (MCMC) simulation was used to obtain a stationary distribution estimating the joint posterior distribution. Parameter convergence was assessed visually by trace plots tracking the iterations of the MCMC simulations.

Data management was conducted in SAS (SAS Institute Inc., version 9.2, Cary, North Carolina). SPSS was utilized for PCA analysis (SPSS Inc., version 17.0.0, Chicago, Illinois). Spatial data management and visualization took place in ArcGIS (Environmental Systems Research Institute, Inc. ArcMap 9.3, Redlands, California). Moran’s I calculations were conducted using GeoDa (Luc Anselin, version 0.9.5-i5), and R statistical software (R Development Core Team, version 2.9.1, Vienna, Austria) with the BRugs package (version 3.0.3) were utilized for all Bayesian analyses.

## Results

**Figure 1:**
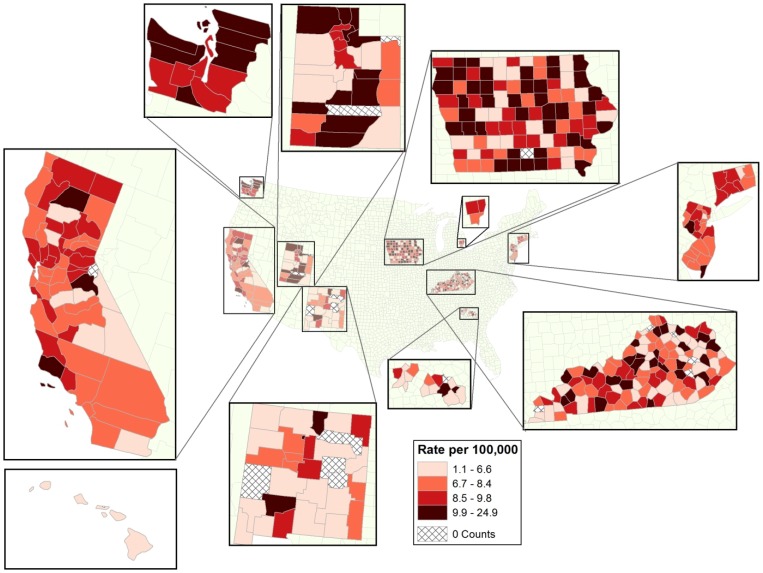
Age-adjusted County Glioma Incidence Rates per 100,000 Within the SEER-17 Study Area, 2000–2006.

A total of 24,230 adult (ages 25+ years) glioma cases were diagnosed in the 17 SEER regions from 2000 through 2006 (see Table 1 for proportions of glioma histologic subtypes). White males had the highest observed average annual, age-adjusted glioma incidence rate (10.47 per 100,000; hereafter, rates are average annual, age-adjusted and are expressed as per 100,000). White females, black males, other race males, black females, and other race females followed with rates of 7.01, 5.21, 4.58, 3.43, and 3.01, respectively. Glioma rates ranged from a low of 1.12 in Socorro County, New Mexico to a high of 24.90 in Pocahontas County, Iowa, with 7.78 as the overall rate for the study region. It appears as if county glioma rates may geographically cluster as high rates tended to be adjacent to one another (Figure 1).

**Figure 2:**
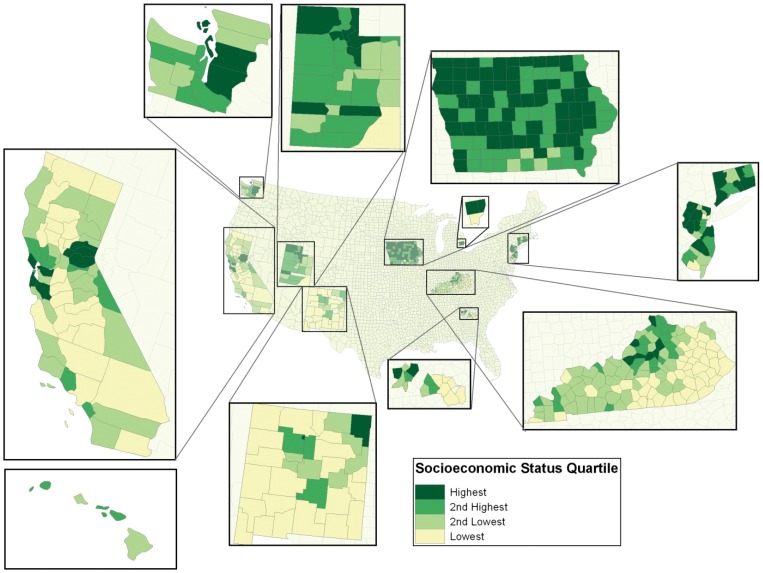
Socioeconomic Scores (2000 U.S. Census Bureau) for Counties Within the SEER 17 Study Area.

PCA of the county SEP-related variables for both the 1990 and 2000 censuses resulted in two factors (Table 2). The factors accounted for 84.4% (factor 1∶44.0% and factor 2∶40.4%) and 85.6% (factor 1∶48.1% and factor 2∶37.5%) of the total 1990 and 2000 county variable variation, respectively. Variables loading most heavily on factor 1 were: percent below 100% FPL; percent less than a high school education; median household income; and percent unemployed. Percent foreign born, percent urban, and median household income loaded heavily on factor 2. Factor 1 is referred to as ‘SEP’ in subsequent analyses because variables loading most heavily were traditional area-based socioeconomic measures (bold variables in table 2) [17], [18]. Factor 1 and factor 2 had zero correlation with one another, as noted above. Factor 2 was excluded in modeling because of its inability to confound SEP [19]. Figure 2 provides visual evidence suggesting that PCA-derived SEP values (year 2000) may geographically cluster. Comparing county maps to one another suggests that county SEP may be associated with county glioma rates as both follow similar geographic distributions.

**Table 2:** PCA Factor Loadings of Six SEP Measures of 404 SEER-17 Counties, 1990 and 2000.

	Factor 1 (“SEP”)		Factor 2	
	1990	2000	1990	2000
% Variance Explained	44.0	48.1	40.4	37.5
% <100% Federal Poverty Line^a^	**0.855**	**0.936**	−0.411	−0.219
%<High School Education^a^	**0.748**	**0.816**	−0.453	−0.328
Median Household Income^a^	−**0.672**	−**0.721**	**0.669**	**0.617**
% Unemployed^a^	**0.922**	**0.898**	0.081	0.183
% Foreign Born^a^	−0.157	−0.090	**0.906**	**0.925**
% Urban	−0.150	−0.111	**0.881**	**0.908**

a Log transformed prior to PCA analysis.

Bold: Variables loading heavily on respective factors.

Abbreviations: PCA, Principal component analysis; SEER, Surveillance Epidemiology and End Results; SEP, Socioeconomic position.

Estimates of the two basic models, model fit statistics, and applicable Moran’s I results are shown in Table 3. Model 1 included individual-level covariates with county-specific random intercepts and county SEP covariates. The model 1, year 1990 estimated rate among young adult, black, females within a typical county was 1.56 (95% CI: 1.45, 1.68) (a typical county is one with an estimated random intercept = 0, or a county with a glioma rate that is similar to that of the overall rate for the entire dataset). Estimated glioma RRs comparing SEP quartiles 4, 3, and 2 to quartile 1 were all statistically insignificant; median estimate and 95% CI: 1.01 (0.95, 1.08), 0.96 (0.90, 1.02), and 1.01 (0.96, 1.05), respectively. The DIC value of model 1, 1990 county SEP, was 11650. County rate error analysis yielded a Moran’s I *P* = 0.017, indicating statistically significant spatial dependence.

**Table 3:** Estimated coefficients, 95% credible intervals, model fit statistics, and Moran’s I values for the two basic models estimating glioma risk using 1990 and 2000 census data.

		Model 1^a^				Model 2^b^				
		1990		2000		1990		2000		
Parameter		RR	(95% CI)	RR	(95% CI)	RR	(95% CI)	RR		(95% CI)
Individual level										
	Intercept^c^	1.56	(1.45,1.68)	1.40	(1.30,1.51)	1.76	(1.43,2.02)	1.42		(1.30,1.55)
Sex										
	Female	Reference		Reference		Reference		Reference		
	Male	1.49	(1.46,1.53)	1.50	(1.46,1.53)	1.50	(1.46,1.53)	1.50	(1.46,1.53)	
Race										
	Black	Reference		Reference		Reference		Reference		
	White	2.01	(1.89,2.14)	2.00	(1.88,2.13)	1.99	(1.87,2.12)	1.99	(1.86,2.11)	
	Other	0.89	(0.82,0.97)	0.88	(0.81,0.96)	0.89	(0.82,0.97)	0.89	(0.81,0.96)	
Age										
	25–44 yrs	Reference		Reference		Reference		Reference		
	45–64 yrs	2.38	(2.30,2.46)	2.38	(2.30,2.46)	2.37	(2.29,2.46)	2.37	(2.29,2.46)	
	65+ yrs	4.87	(4.71,5.04)	4.87	(4.71,5.04)	4.86	(4.69,5.02)	4.86	(4.69,5.03)	
County level										
Socioeconomic Quartile										
	Lowest	Reference		Reference		Reference		Reference		
	2nd Lowest	1.01	(0.95,1.08)	1.13	(1.08,1.19)	0.85	(0.73,1.00)	1.10	(1.02,1.19)	
	2nd Highest	0.96	(0.90,1.02)	1.15	(1.09,1.21)	0.83	(0.69,1.08)	1.11	(1.02,1.20)	
	Highest	1.01	(0.96,1.05)	1.19	(1.13,1.25)	0.81	(0.61,1.20)	1.14	(1.05,1.23)	
DIC		11650		11620		11630		11620		
Moran’s I (P-value)		0.073	(0.017)	0.066	(0.021)	0.010	(0.357)	−0.041		(0.117)

a Individual level covariates with a county random intercept+socioeconomic county covariates in a second level.

b Model 1+ conditionally autoregressive prior on random intercepts.

c Model intercepts may be interpreted as estimated rates per 100,000 among the young adult, black, female subgroup for a ‘typical’ county (a county with estimated random intercept = 0).

Abbreviations: CI, Credible Interval; DIC, Deviance Information Criterion; RR, Rate Ratio.

Model 1, year 2000, estimated a typical intercept of 1.40 (95% CI: 1.30, 1.51). Individual level RR estimates differed negligibly between both decennial census models. Those individuals residing in counties of the fourth, third and second SEP quartiles had estimated rates that were 1.19 (95% CI: 1.13, 1.25), 1.15 (95% CI: 1.09, 1.21), and 1.13 (95% CI: 1.08, 1.19) times that of individuals in the first quartile of SEP, respectively, adjusting for age, race, and sex. Model 1, year 2000, DIC was 11620. Moran’s I of county rate error yielded *P* = 0.021.

Model 2 followed the framework of model 1 while spatially structuring the random intercepts according to a CAR prior distribution. As in model 1, individual-level RRs did not change between models using the two census years. The estimated intercept of model 2, year 1990 SEP, was 1.76 (95% CI: 1.43, 2.02). Each of the SEP quartiles had 95% CIs that included the null; median estimate and 95% CI: 0.85 (0.73, 1.00), 0.83 (0.69, 1.08), and 0.81 (0.61, 1.20), for rate comparisons between quartiles 4, 3, and 2, with quartile 1, respectively. Model 2, year 1990 DIC was 11630. Moran’s I of county rate error yielded *P* = 0.357, suggesting spatial independence.

Model 2, year 2000 county SEP, estimated a typical intercept of 1.42 (95% CI: 1.30, 1.55). Those individuals residing in counties of the fourth, third and second SEP quartiles at time of diagnosis had estimated rates that were 1.14 (95% CI: 1.05, 1.23), 1.11 (95% CI: 1.02, 1.20), and 1.10 (95% CI: 1.02, 1.19) times that of individuals in the first quartile of SEP, respectively, adjusting for age, race, and sex. Rate ratios of the first, second and third quartiles (versus fourth) for percent unemployed, percent less than a high school education, and percent impoverished were generally similar in magnitude, trend and statistical significance to the SEP component (between 1.17 to 1.05, respectively). Rate ratios of the second, third and fourth quartiles (versus first) for median household income were lower compared to the SEP component, did not follow a clear pattern, and were not statistically significant (between 0.95 to 1.05, respectively) (results not shown in tables). Model 2, year 2000 model DIC was 11620. Moran’s I of county rate error yielded *P = *0.117.

Due to superior fit, year 2000 census data was used for confounding and effect modification evaluation. There was only slight (<7%) confounding of the SEP-glioma relationship by individual-level factors. Cross-level effect modification was examined by stratifying counties on SEP quartiles and refitting models including individual-level covariates with county random intercepts. All 95% CIs overlapped across SEP strata. Glioma RRs for whites monotonically increased with increased SEP strata; median and 95% CI: 1.86 (1.68, 2.05), 2.02 (1.80, 2.27), 2.14 (1.84, 2.51), and 2.24 (1.92, 2.61) for SEP quartiles 1, 2, 3, 4, respectively. RR estimates comparing middle-aged to young adults stratified by SEP quartiles were 2.52 (2.36, 2.68), 2.35 (2.19, 2.52), 2.43 (2.26, 2.60), and 2.24 (2.10, 2.40). RRs for males, other races, and elderly overlapped appreciably between SEP strata, suggesting no effect modification.

**Figure 3:**
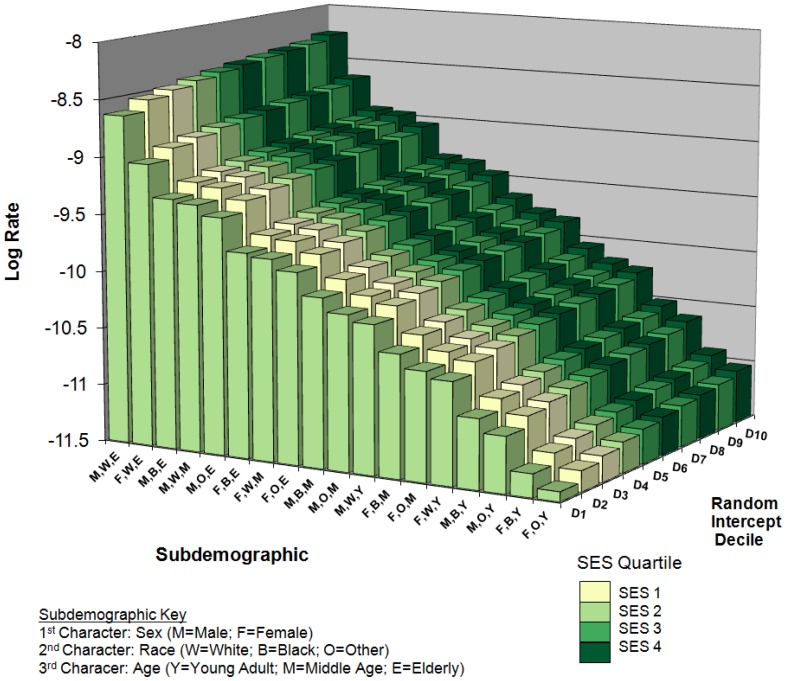
Bar Chart Summary of Estimated Log Rates by Subdemographic and County Random Intercept Decile^a,b^. ^ a^Unstandardized rates produced from model 2, year 2000 estimates. ^b^Deciles (D1–D10) represent the median random intercept values within each decile of random intercepts.

Figure 3 is a visual summary of estimates derived from model 2, year 2000, enabling the simultaneous examination of the explained individual-level and total county-level sources of variation. Random intercepts were chosen based on their rank-ordered position among all 404 county random intercepts. Each of the 10 example counties represented the median random intercept value among each of the 10 random intercept deciles (D) (e.g., 20^th^, 61^st^, 101^st^, …, 384^th^, for D1, D2, D3, …, D10). Bars were shaded according to county SEP quartile. The majority of variation explaining glioma rates occurred among the demographic subgroups, as indicated by the larger range of log rate values across the X-axis. The increase in glioma rates as a function of increased county random intercept was less pronounced, but still appreciable.

## Discussion

These analyses demonstrate a modest, yet statistically significant, association between county-level SEP and individual glioma incidence within the large and heterogeneous group of 17 SEER cancer registries. The best fitting model demonstrated a glioma incidence rate among those living in respective counties of the fourth, third and second highest quartiles of SEP that was 1.14, 1.11, and 1.10 times the rate of those living in first quartile counties. These estimates were statistically adjusted for individual sex, race, and age, despite no appreciable confounding by these factors. Despite relatively small variability in county glioma rates, this variability was statistically significantly associated with county SEP.

Results from three reports of glioma risk associated with similarly measured SEP constructs are consistent with results from the present analyses. Recently, Wigertz, et al. reported that those with family incomes in the highest quartile had 1.5 times the odds of glioma (95% CI: 1.1, 2.1) compared to those in the lowest quartile, adjusted for sex, age, and geographical region [4]. Although the confidence limits of ORs reported by Wigertz, et al. overlap with those reported here, the difference in magnitude may be due to differences in SEP characterization accuracy; our county SEP measures are less accurate than are those based on individual income and education. Chakrabarti, et al. estimated glioblastoma mutliforme RRs that are similar to those estimated here; those living in census tracts of the highest SEP tertile had 1.3 times (95% CI: 1.2, 1.4) the rate compared to those in census tracts of the lowest tertile [3]. However, despite the use of area-based measures, a single-level regression framework was employed, underestimating standard errors associated with census tract-based SEP estimates [7].

Both models utilizing 1990 decennial census information resulted in null associations between SEP and glioma as well as lower estimates of model fit compared to analogous models utilizing data from the 2000 census. Models utilizing the 2000 census data demonstrated monotonic relationships between county SEP and glioma. If these associations are true, insight could be gathered into the mechanisms giving rise to the perceived associations between glioma incidence and area SEP.

Temporally, the mechanism driving the association between year 2000 county SEP and individual glioma diminishes when county SEP from a decade earlier is used. This can be explained by a number of processes taking place, each providing a different picture of the SEP-glioma relationship. First, a specific latency period may explain the results. Attempts to model glioma by the identical construct at latencies greater than 10 years may result in null effects because the window of heightened risk due to exposure has passed. Second, the window of heightened risk relating county SEP to glioma could indeed be present for periods longer than 10 years, but the length of time between the cross sectional exposure measure and glioma diagnosis may lead to exposure misclassification bias, due to factors such as migration of glioma cases across counties of varying SEP. Assuming that this misclassification was non-differential (i.e., a similar pattern of glioma cases moved from high to low counties as did those moving from low to high) and that the county SEP represents an integral or structural construct, estimates would then be biased towards the null (models 1 and 2, 1990 census data) [20], [21]. The magnitude of this possible bias is clearly immeasurable without knowledge of residential histories of glioma cases. A third situation may arise in which county SEP ranks change from the 1990 to 2000 census. Indeed, counties did move in and out of SEP quartiles comparing 1990 and 2000 census data. Approximately 15.5% of the 404 counties moved from a higher to lower SEP quartile, 71.0% remained in the same, and 13.5% moved from lower to higher SEP quartiles. Moreover, differential misclassification may exist as counties with positive random intercepts have patterns of SEP change that differ from those with negative random intercepts. Of negative random intercept counties, 12.4%, 80.9% or 6.7% increased, stayed the same, or decreased SEP quartiles, respectively, from the 1990 to 2000 census. Comparatively, 16.5%, 68.3%, or 15.2% positive intercept counties increased, stayed the same, or decreased SEP quartiles. This type of exposure misclassification may be biasing the 1990 county SEP model estimate either direction from the null [19]. Therefore, one of the conditions for differential misclassification of 1990 county SEP has been demonstrated to exist. However, assessment of magnitude and direction of this bias are not possible with these data.

Cross level effect modification may be occurring, as some race- and age-related glioma RRs varied when stratified by county SEP quartile. For example, RRs comparing whites to blacks increased monotonically from 1.86 to 2.02, 2.14, and 2.24 for the respective stratifications of counties in SEP quartiles 1, 2, 3, and 4, albeit, with moderate overlap of confidence intervals. This may suggest that the rate of glioma comparing whites to blacks varies depending on county SEP, with a widening of the glioma disparity between whites and blacks in counties with higher SEP. Similarly, the glioma RRs vary appreciably comparing middle-aged adults to young adults when stratified on SEP quartile; however, the trend was not monotonic (glioma RR for SEP quartiles 1–4; 2.52, 2.35, 2.43, and 2.24, respectively) and confidence intervals of the 1^st^ and 4^th^ SEP quartiles slightly overlapped, suggesting that the ratio of glioma incidence rates comparing middle-aged to young adults among counties of the lowest SEP quartile is larger than the same comparison in counties of the highest SEP quartile, adjusting for sex and race. Further analyses are needed to confirm the possibility of cross-level modification as these estimates may be too unstable and were conducted as part of secondary analyses.

Group-level factors that are contextual can be described as an “aggregate of attributes measured at the individual level.” [21] County-level SEP is a construct that has origins in the measurement of another set of lower-level attributes. Discussions on the topic are rooted in ecologic fallacy and have noted that the group construct – and association with the outcome – is *rarely* simply an aggregate of the individual analogs and its association with that outcome [22]–[25].

Conclusions based on a true effect would be more tenable had information on individual-level SEP been available and included in the present analysis. After statistical adjustment for individual-level SEP, significant county-level SEP estimates arguably could *not* be the result of SEP relationships occurring at the individual-level. Our results may have been due to a direct psychosocial effect [26] or an integral/structural [21] phenomenon which county SEP proxied such as healthcare accessibility. It is also possible that our results are related to previous literature demonstrating links between glioma risk and occupation. Higher county SEP may be related to higher percentages of employment in white-collar and professional occupations. Support for this may be found in the positive associations between education, employment and glioma rates. However, lack of individual-level data and any substantial theory mechanistically explaining these observed associations between SEP gradients – individual or group – and glioma, precludes any conclusions pertaining to the true magnitude and public health significance of these associations.

Our case definition included many glioma subtypes which exhibit disparate characteristics. Glioma researchers have noted flaws with heterogeneously classifying disease [27]–[29]. The inability to examine specific glioma subtypes may be a slight weakness; however, the low incidence of glioma and Poisson nature of these analyses prohibited division of glioma into more homogenous histologic subtypes. It should be noted that the estimated glioma associations may have arisen from a process similarly affecting all glioma subtypes, or a representation of the associations belonging to the most dominant glioma subtype within this group (i.e., glioblastoma).

The relatively small proportion of explained variability due to county effects underscores the possibility that the observed SEP-glioma association could be due to unmeasured individual-level confounding. This finding was previously suggested by other researchers who noted the relatively small between group variability of brain tumor rates [28], [30]. Ionizing radiation and genetic mutations are the only definitive “causes” of glioma [27]–[29]. Including all available individual factors that could possibly confound the glioma-SEP relationship attenuated the county SEP estimates between 2.6%–6.5%. The relatively tight confidence bounds of these estimates about the posterior median value were widened close to the null when proper control of error spatial dependence was made. Despite this preponderance towards the null with adjustment, one must keep in mind that if the associations between SEP (either individual or area-based) and glioma result from confounding, the confounder must satisfy the conditions of a confounder: 1) be related to glioma incidence, 2) be related to SEP, and 3) not mediate the SEP-glioma relationship [19]. Moreover, this confounding association would seemingly cut across multiple geographic scales as the SEP-glioma association has been reported at the national [28]–[30], postal code [31], census tract [3], individual [4], [32], and now county levels.

The leading candidates serving as a common confounder to these multi-scale associations are differential diagnosis and reporting of glioma [27]–[30], [33], [34]. Glioma may be associated with SEP due to increased access to healthcare; those individuals of higher SEP are able to access healthcare more readily, leading to increased incidental diagnoses of glioma. The lack of individual-level information on health insurance status, healthcare utilization, or other measures related to healthcare access limits the ability to ascribe causal inference to the SEP-glioma relationship. One study identified the disproportionate diagnoses of glioblastomas in Connecticut compared to other SEER registries [35]. Diagnostic bias due to incidental tumor discovery has also been noted for diagnoses of meningioma [36], a tumor that does not appear to be associated with SEP [4], [27], [31], [37]. It should be noted, however, that there has been no rigorous analysis of SEP and meningioma rates, and such an analysis may further inform the differences between meningioma and glioma in terms of association with SEP.

Ascertainment bias caused by a lack of universal standards in cancer diagnosis and reporting strategies by both physicians and registries is another possible explanation of the SEP-glioma association. However, as Surawicz [34] points out, this bias may have less of a detrimental effect on cancers like glioma. Malignant glioma are characterized by high case completion percentages compared to other cancer types [38], [39]. Percent of complete cases is a measure routinely calculated by cancer registries that estimates the number of unreported cases which have not been underreported as compared to national figures [38]. The high quality and completeness of the SEER registry decreases the possibility of ascertainment bias by cancer registries..

The strengths of this analysis lie in the methodologic rigor taken to yield the least biased and most accurate estimates with the available high quality SEER registry data. Much of the previous research suggesting or reporting on similar SEP-glioma associations either did so using crude proxies [5], [40], [41], as a secondary aim [4], or in a methodologically biased fashion by conflating data of different geographic scales and making inference on the individual level [3], [31], [37] – providing the possibility for ecologic fallacy and/or underestimating standard errors [7], [24]. This was the first glioma-SEP study utilizing a multilevel model to explicitly investigate the possibility of previously suggested between group variability [28]–[30], investigating a time lag between SEP and glioma, investigating error spatial dependence, and reporting on possible cross-level effect modification.

## Supporting Information

Equation S1 (DOC)
