# Supplementary material for: Area-Based Socioeconomic Position and Adult Glioma: A Hierarchical Analysis of Surveillance Epidemiology and End Results Data
Source: PLoS One. 2013 Apr 9;8(4):e60910. doi: 10.1371/journal.pone.0060910 (PMC3622005; doi:10.1371/journal.pone.0060910)
Supplement: Equation S1 — (DOC) [file pone.0060910.s001.doc]

Equation S1.

The model with both 1st and 2nd level covariates (demographic subgroupings and county-level SEP components, respectively) can be notationally represented as:

where within the individual level (superscript ‘I’) *cij* is theaggregate glioma count for the given subgroup *i* within county *j*; *r* is the subgroup population (serving as an offset); *β0* is the overall intercept of the model; *β1­* - *β5­*are theregression coefficients of variables *X1 - X5*; *X1* is the variable male (female is referent)*,* X2 is the variable white (black is referent),X3 is the variable other (black is referent)*,* X4 is the variable middle-age (young adult is referent)*,*X5 is the variable elderly (young adult is referent). Within the county level (superscript ‘C’) *λj* is thecounty-level effect, *β1­*, *β2,…, βn­* are the regression coefficients of the subsequent PCA-derived SEP components, and *τ2* is the variance of the county-level effects. Glioma rate ratios can be calculated from resulting model estimates by exponentiating the regression coefficients – exponentiation of any intercept-associated estimate (e.g., *βI0* or [*βI0 +j*]) simply yields an unstandardized rate.
